# Prognostic impact of gene alterations via homologous recombination DNA repair gene alteration status in pancreatic ductal adenocarcinoma

**DOI:** 10.3389/fmed.2025.1570731

**Published:** 2025-08-25

**Authors:** Yusuke Kawanaka, Chiaki Inagaki, Masaki Okura, Seiichiro Mitani, Takayuki Takahama, Kimio Yonesaka, Yasutaka Chiba, Kazuhiko Nakagawa, Hisato Kawakami, Hidetoshi Hayashi

**Affiliations:** ^1^Department of Medical Oncology, Kindai University Faculty of Medicine, Osakasayama, Japan; ^2^Genome Medical Center, Kindai University Hospital, Osakasayama, Japan; ^3^Clinical Research Center, Kindai University Hospital Ohno-Higashi, Osakasayama, Japan; ^4^Cancer Center, Kindai University Hospital, Osakasayama, Japan; ^5^Department of Clinical Oncology, Tohoku University Graduate School of Medicine, Sendai, Japan

**Keywords:** homologous recombination DNA repair, pancreatic ductal adenocarcinoma, comprehensive genomic profiling, real-world data, chemotherapy

## Abstract

**Background:**

Pancreatic ductal adenocarcinoma (PDAC) is one of the most lethal malignancies, with limited treatment options and poor prognosis. Recent advances in cancer genomic analysis enable the identification of actionable gene alterations, opening new opportunities for personalized therapy. Among these, homologous recombination DNA repair (HRR) gene alterations are associated with distinct biological behavior, favorable prognosis, and increased sensitivity to platinum-based chemotherapy. However, the prognostic impact of coexisting mutations in key driver genes—*KRAS*, *TP53*, *CDKN2A*, and *SMAD4*—within HRR-altered PDAC remains poorly understood.

**Methods:**

We retrospectively analyzed PDAC patients who underwent genomic profiling testing with FoundationOne® CDx between June 2019 and December 2021 through the Center for Cancer Genomics and Advanced Therapeutics (C-CAT) database. We compared the prevalence and prognostic significance of key gene alterations between HRR-altered and HRR–wild-type (WT) tumors.

**Results:**

Of 2,381 PDAC patients, 274 (11.5%) harbored HRR alterations. These patients showed significantly longer overall survival (OS) than those with HRR-WT tumors (HR = 0.66, *p* = 0.002). The frequencies of *KRAS*, *TP53*, and *CDKN2A* mutations were less frequent in HRR-altered tumors. *TP53* mutation was independently associated with poorer OS across both HRR subgroups, while *CDKN2A* alteration was a poor prognostic factor in HRR-WT tumors. Interestingly, *SMAD4* alteration was linked to improved survival in the HRR-altered group.

**Conclusion:**

HRR-altered PDAC has a distinct genomic profile and is associated with a favorable prognosis. Our findings demonstrate that coexisting alterations are significant prognostic factors in both HRR-altered and HRR–wild-type tumors. These results highlight the clinical relevance of incorporating comprehensive genomic profiling into routine care to stratify patient prognosis better and inform individualized treatment strategies in PDAC.

## Introduction

Pancreatic ductal adenocarcinoma (PDAC) is the sixth leading cause of cancer-related deaths worldwide (1). The prognosis of metastatic and advanced PDAC remains poor, with a 5-year survival rate of less than 10% (2). The integration of molecular profiling, including next-generation sequencing (NGS), into routine clinical practice has significantly advanced our understanding of genetic alterations associated with PDAC. *KRAS*, *TP53*, *CDKN2A*, and *SMAD4* are the most frequently altered genes in PDAC. Although these gene alterations contribute to aggressive tumor characteristics and lead to treatment resistance and poor prognosis (3–6), they have remained undruggable for decades. However, recent scientific advances have enabled the targeting of these gene alterations. For example, structural and biochemical analyses can now be used to design drugs targeting *KRAS* mutations and p53 (7, 8). Moreover, rapid progress in proteolysis-targeting chimera (PROTAC) technology has provided a new strategy for targeting activated KRAS proteins (9), providing hope for improving the prognosis of PDAC.

Homologous recombination DNA repair (HRR) gene alterations have been identified in 14–16.5% of PDAC patients (10, 11), and these patients have a more favorable prognosis than those with wild-type (WT) (12, 13). Further, PDAC patients with HRR gene mutations demonstrate greater sensitivity to platinum-based therapies than do those with HRR-WT PDAC (14). PARP inhibitors have been evaluated in PDAC patients with HRR gene mutations based on the concept of synthetic lethality (15); however, their efficacy is limited to germline *BRCA1/2* mutations in maintenance therapy after platinum-based treatment (16). With the recent progress in molecularly targeted therapies for PDAC, a comprehensive understanding of the genetic landscape and its influence on survival outcomes has become an important area of investigation. Previous studies have noted that the mutation frequencies of *KRAS*, *TP53*, and *CDKN2A* in PDAC differ depending on the presence or absence of HRR gene alterations (17, 18); however, the prognostic significance of these genetic alterations when stratified by HRR gene alteration status has not yet been explored.

Thus, this study aimed to evaluate the prevalence and prognostic impact of coexisting gene alterations according to the HRR gene alteration status in PDAC. Toward this goal, we analyzed the data of patients with PDAC registered in the Center for Cancer Genomics and Advanced Therapeutics (C-CAT) (19), the largest real-world comprehensive genomic profiling registry in Japan.

## Methods

### Ethical approval

This study was approved by the Medical Ethics Review Committee of Kindai University of Medicine (Approval Number: R05-029) and the C-CAT review board (C-CAT Control Number: CDU2023-021 N) and was conducted according to the tenets of the Declaration of Helsinki.

### Study design and patients

This retrospective observational study was conducted using data from the C-CAT database (19), a repository of clinical and genomic information on Japanese patients with cancer who underwent genomic profiling tests as part of the insurance system. This study focused on patients with pancreatic cancer who underwent Foundation One® CDx (F1CDx) testing (Foundation Medicine Inc., Cambridge, USA) (20). All reported pathogenic and likely pathogenic mutations met the quality control criteria defined by Foundation Medicine. The limit of detection (LOD) of F1CDx varies depending on the type of variant. The LOD ranges from 1.8 to 7.9% for base substitutions and from 7.1 to 11.7% for insertions and deletions (indels) (20, 21). All patients registered between June 2019 and December 2021 were included whose clinical and genomic data available at the time of the data update in April 2022. PDAC patients were identified as those documented with pancreatic adenocarcinoma according to the OncoTree cancer classification platform (November 2, 2021), within the C-CAT database (22). Briefly, the C-CAT provides the following information: age, sex, Eastern Cooperative Oncology Group performance status (ECOG PS), smoking history, drinking habit, cancer type, pathological diagnosis, site of metastasis, site of the specimen used for genomic testing, type of specimen used for genomic testing, date of death, last confirmed date of survival, and information related to chemotherapy (e.g., regimens, first and last date of administration, best response, date of progression, and serious adverse events). Genomic information provided by C-CAT includes genetic alterations detected, allele frequencies, copy number (CN), and pathogenicity.

### Outcomes

Clinical and genomic data were extracted on January 2024. HRR-altered PDAC patients was identified as those with pathogenic or likely pathogenic alterations in the following genes: *ATM*, *BAP1*, *BARD1*, *BLM*, *BRCA1*, *BRCA2*, *BRIP1*, *CHEK2*, *FAM175A*, *FANCA*, *FANCC*, *NBN*, *PALB2*, *RAD50*, *RAD51*, *RAD51C*, and *RTELI.* The prevalence of major pathogenic alterations in the frequently mutated genes *KRAS*, *TP53*, *SMAD4*, and *CDKN2A*, referred to as the “Big 4” driver mutations, was then compared between HRR-altered and HRR-WT PDAC patients. The prevalences of mutations and CN loss were analyzed separately. Patients with mutations and CN loss in the same gene (one patient with *CDKN2A* mutations and two patients with *SMAD4* mutations) were excluded. Treatment outcomes were evaluated in patients who received the folinic acid, fluorouracil, irinotecan hydrochloride, and oxaliplatin (FFX) regimen including modified FFX or the gemcitabine and nab-paclitaxel (GA) regimen in the first-line setting. Patients who received perioperative chemotherapy were excluded. Overall survival (OS) and time to treatment failure (TTF) were analyzed. OS was defined as the duration (days) from the date of first-line chemotherapy initiation for locally advanced or metastatic PDAC to the date of death or the last confirmed date of survival. TTF was defined as the duration from the start of treatment to the date of treatment discontinuation or death from any cause. Progression free survival, overall response rate, and adverse events were not evaluated in this study because relevant data were not available in the C-CAT registry.

### Statistical analysis

The prognostic significance of HRR gene alteration status was evaluated with respect to OS and TTF. The HRR-altered group was compared to the HRR wild-type (HRR-WT) group using Kaplan–Meier method with the log-rank test. Additionally, interactions between *TP53*, *CDKN2A*, *KRAS* and *SMAD4* alteration status and OS were analyzed within each HRR group. Univariate and multivariate analyses were performed by using the Cox proportional hazard model. The prevalence of these genetic alterations within the two HRR groups was summarized, and differences between the groups were calculated. We reported *p* values in addition to confidence intervals and statistical significance was set at *p* < 0.05; however, there were not to be interpreted as hypothesis tests. The results should be interpreted with caution.

All statistical analyses were performed using EZR version 4.3.1 (Saitama Medical Center, Jichi Medical University, Saitama, Japan) (23), and using GraphPad Prism version 10.2.2 for Windows (GraphPad Software, Boston, Massachusetts USA, www.graphpad.com).

## Results

### Clinical and molecular characteristics based on HRR gene alteration status

**Figure 1:**
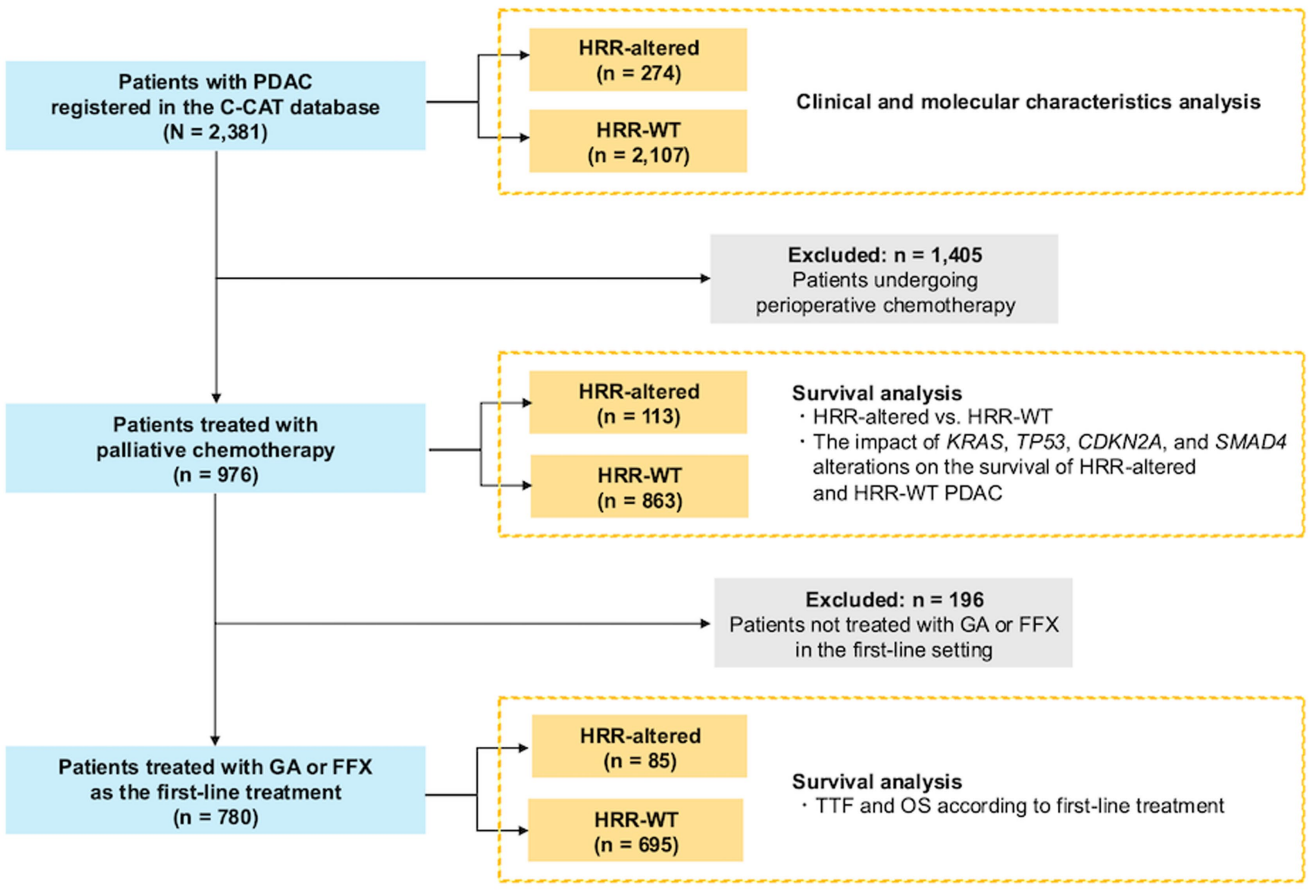
CONSORT diagram of the study.

Among the 2,381 patients with PDAC, 274 patients (11.5%) had HRR alteration (Figure 1). The most frequently altered HRR genes were *ATM* (*n* = 87, 32%) and *BRCA2* (*n* = 87, 32%), followed by *PALB2* (*n* = 40, 15%) (Supplementary Figure 1). The clinical characteristics of the patients in the HRR-altered and HRR-WT groups are shown in Table 1. The median patient age was 65 years (range, 30–83 years) in the HRR-altered group and 67 years (range, 26–88 years) in the HRR-WT group. The median (range) ECOG PS was 0 (0–3) in the HRR-altered group and 0 (0–3) in the HRR-WT group. Sex distribution and the number of metastatic sites were comparable between the two groups. With respect to the prevalence of gene alterations, *KRAS* mutations (77% vs. 97%, *p* < 0.001), *TP53* mutations (48% vs. 79%, *p* < 0.001), and *CDKN2A* mutations (10% vs. 19%, *p* < 0.001) were significantly less prevalent in the HRR-altered group than in the HRR-WT group (Table 2).

**Table 1:** Patient characteristics by HRR alteration status.

Characteristic	HRR-altered group	HRR-WT group
	*n* = 274	*n* = **2,107**
Age at registration, years		
median (range)	65 (30–83)	67 (26–88)
Sex, *n* (%)		
Female	124 (45)	933 (44)
Male	150 (55)	1,174 (56)
Unknown	0 (0)	0 (0)
Smoking, *n* (%)		
Yes	127 (46)	1,001 (48)
No	132 (48)	1,014 (48)
Unknown	15 (5)	92 (4)
Heavy alcohol consumption, *n* (%)		
Yes	35 (13)	253 (12)
No	206 (75)	1,638 (78)
Unknown	33 (12)	216 (10)
ECOG PS, *n* (%)		
0–1	259 (95)	2,006 (95)
≥ 2	6 (2)	38 (2)
Unknown	9 (3)	63 (3)
Metastatic sites, *n* (%)		
1	143 (52)	1,165 (55)
≥ 2	100 (36)	689 (33)
Unknown	31 (11)	253 (12)
Sampling methods, *n* (%)		
Surgery	137 (50)	1,146 (54)
Biopsy	135 (49)	936 (44)
Unknown	2 (1)	25 (1)

HRR, homologous recombination DNA repair; WT, wild type; ECOG PS, Eastern Cooperative Oncology Group performance status.

**Table 2:** Prevalence of *KRAS*, *TP53*, *SMAD4*, and *CDKN2A* gene alterations based on HRR gene alteration status.

Gene alteration	Status	HRR-altered group (*n* = 274)	HRR-WT group (*n* = 2,107)	Percentage difference (%)	*p* Value
*KRAS* mutation, *n* (%)	+	212 (77)	2,039 (97)	20	< 0.001
	−	62 (23)	68 (3)	−	
*TP53* mutation, *n* (%)	+	132 (48)	1,656 (79)	31	< 0.001
	−	142 (52)	451 (21)	−	
*CDKN2A* mutation, *n* (%)	+	28 (10)	410 (19)	9	< 0.001
	−	246 (90)	1,697 (81)	−	
*CDKN2A* Loss, *n* (%)	+	84 (31)	684 (32)	1	0.583
	−	190 (69)	1,423 (68)	−	
*SMAD4* mutation, *n* (%)	+	54 (20)	421 (20)	0	1.000
	−	220 (80)	1,686 (80)	−	
*SMAD4* Loss, *n* (%)	+	13 (5)	166 (8)	3	0.085
	−	261 (95)	1,941 (92)	−	

HRR, homologous recombination DNA repair; WT, wild type.

Fisher’s exact test was used to compare the proportions between the groups.

### Clinical outcomes according to HRR gene alteration status

**Figure 2:**
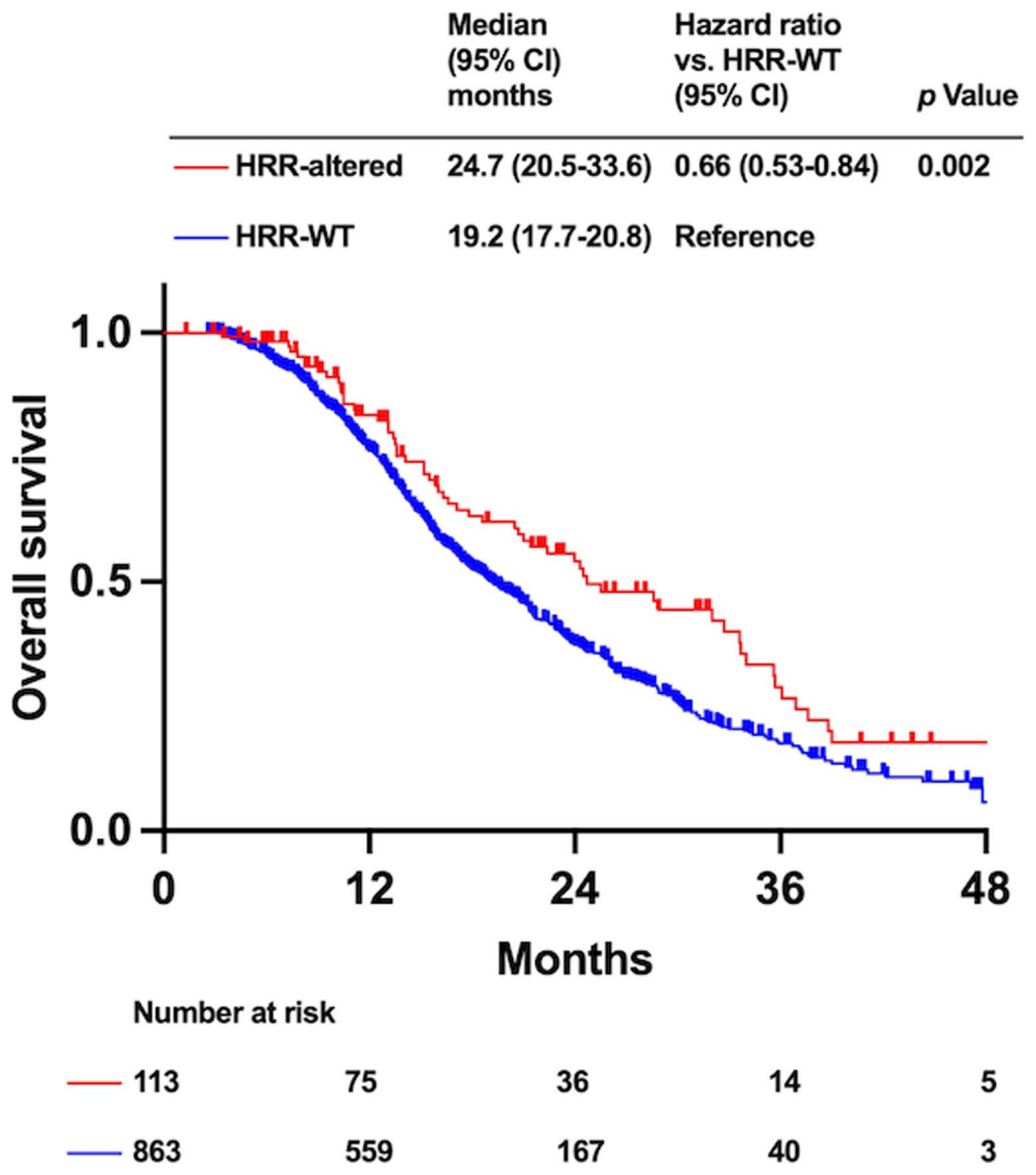
Kaplan–Meier analysis of overall survival (OS) based on homologous recombination DNA repair (HRR) gene alteration status.

A total of 976 patients who were treated with chemotherapy only for palliative intent and had available survival data were included in the survival analysis (Figure 1). The clinical characteristics of the patients are shown in Supplementary Table 1. In total, 113 and 863 patients had HRR-altered and HRR-WT PDAC. Patients with HRR-altered PDAC tend to have a better prognosis and obtain greater survival benefits from platinum-based agents than HRR-WT patients. Therefore, we analyzed survival outcomes based on the HRR gene alteration status. The results showed that OS was significantly better in the HRR-altered group than in the HRR-WT group (median OS: 24.7 months [95% CI: 20.5–33.6 months] vs. 19.2 months [95% CI: 17.7–20.8 months], *p* = 0.002; Figure 2).

Meanwhile, 308 patients who received FFX and 472 patients who received GA as the first-line therapy were included in the comparison of TTF and OS between the HRR-altered and HRR-WT groups. The clinical characteristics of the patients are shown in Supplementary Table 2. In patients treated with FFX as the first-line therapy, TTF was significantly prolonged in the HRR-altered group (median TTF: 7.8 months [95% CI: 5.5–9.6 months] vs. 5.4 months [95% CI: 4.3–5.8 months], *p* = 0.012; Supplementary Figure 2A). Although OS was not significantly different, there was a favorable trend in the HRR-altered group (median OS: 24.3 months [95% CI: 13.5–36.1 months] vs. 16.8 months [95% CI: 15.5–19.5 months], *p* = 0.100; Supplementary Figure 2B). In patients treated with GA as the first-line therapy, both TTF (median: 5.4 months [95% CI: 4.4–6.9 months] vs. 5.2 months [95% CI: 4.7–6.0 months], *p* = 0.859) and OS (median: 24.5 months [95% CI: 17.1–38.8 months] vs. 20.8 months [95% CI: 18.8–22.9 months], *p* = 0.094) were comparable according to the HRR gene alteration status (Supplementary Figures 3A,B).

### Distinct impact of KRAS, TP53, SMAD4, and CDKN2A gene alterations by HRR alteration status

We investigated the impact of *KRAS*, *TP53*, *SMAD4*, and *CDKN2A* alterations on the survival of HRR-altered and HRR-WT PDAC, respectively.

**Figure 3:**
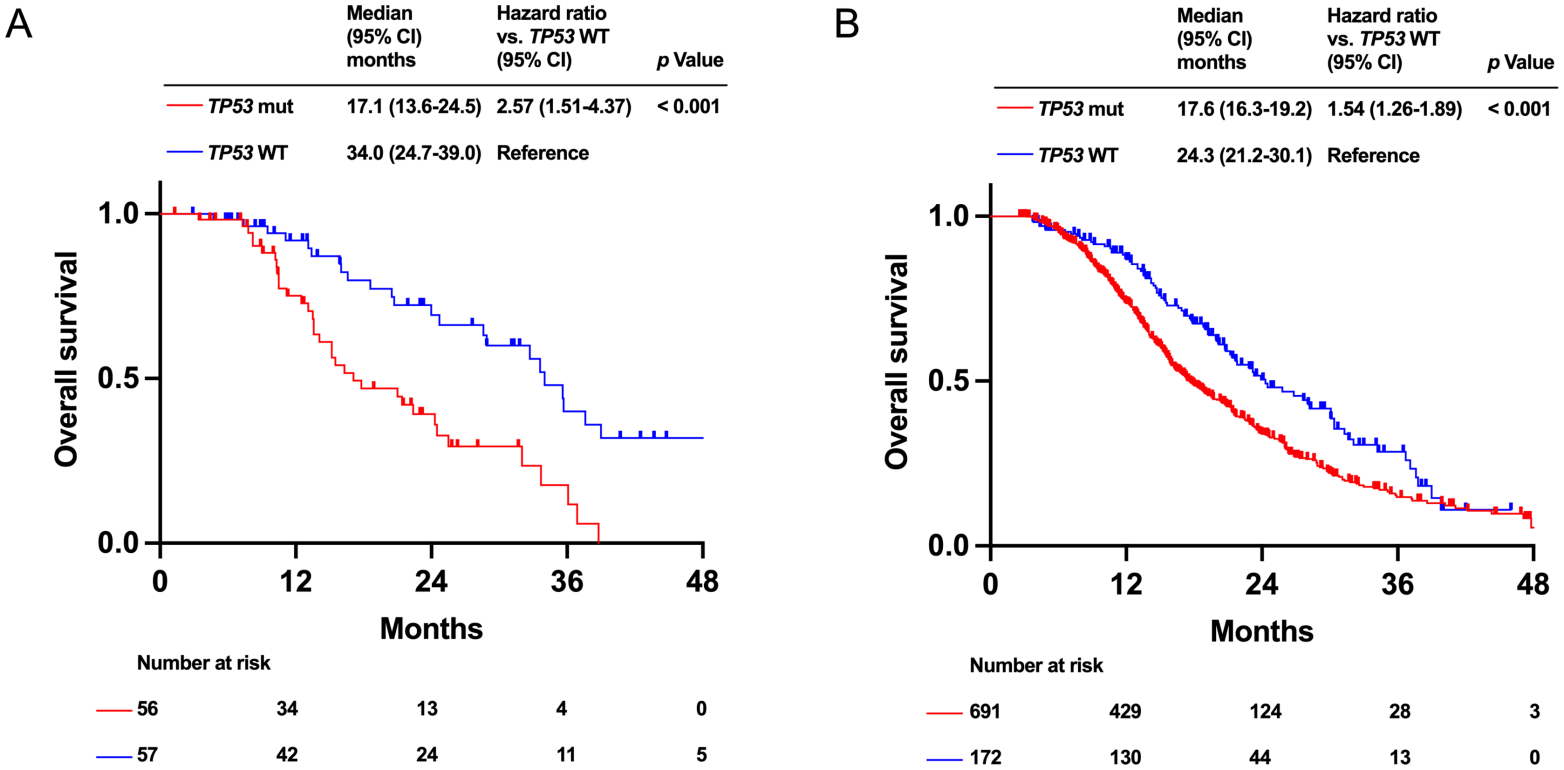
Kaplan–Meier analysis of overall survival (OS) by *TP53* mutation status in PDAC patients. Data for the homologous recombination DNA repair (HRR)-altered group **(A)** and the HRR wild-type (WT) group **(B)** are shown.

**Figure 4:**
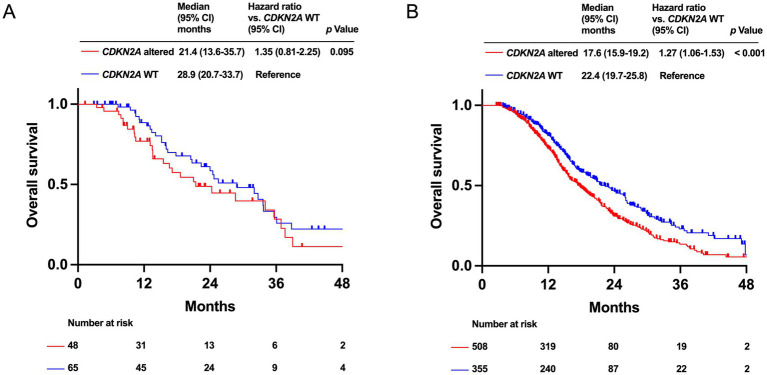
Kaplan–Meier analysis of overall survival (OS) by *CDKN2A* alteration status in PDAC patients. Data for the homologous recombination DNA repair (HRR)-altered group **(A)** and the HRR wild-type (WT) group **(B)** are shown.

Patients with *TP53* mutation had significantly worse prognosis in both the HRR-altered (median OS: 17.1 months [95% CI: 13.6–24.5 months] vs. 34.0 months [95% CI: 24.7–39.0 months], *p* < 0.001) and HRR-WT (median OS: 17.6 months [95% CI: 16.3–19.2 months] vs. 24.3 months [95% CI: 21.2–30.1 months], *p* < 0.001) groups (Figures 3A,B). In the HRR-altered group, patients with *CDKN2A* alteration tended to have a poor OS compared to *CDKN2A* WT patients (median OS: 21.4 months [95% CI: 13.6–35.7 months] vs. 28.9 months [95% CI: 20.7–33.7 months], *p* = 0.095). In the HRR-WT group, patients with *CDKN2A* alteration had significantly shorter OS compared to patients with *CDKN2A* WT (median OS: 17.6 months [95% CI: 15.9–19.2 months] vs. 22.4 months [95% CI: 19.7–25.8 months], *p* < 0.001; Figures 4A,B).

**Figure 5:**
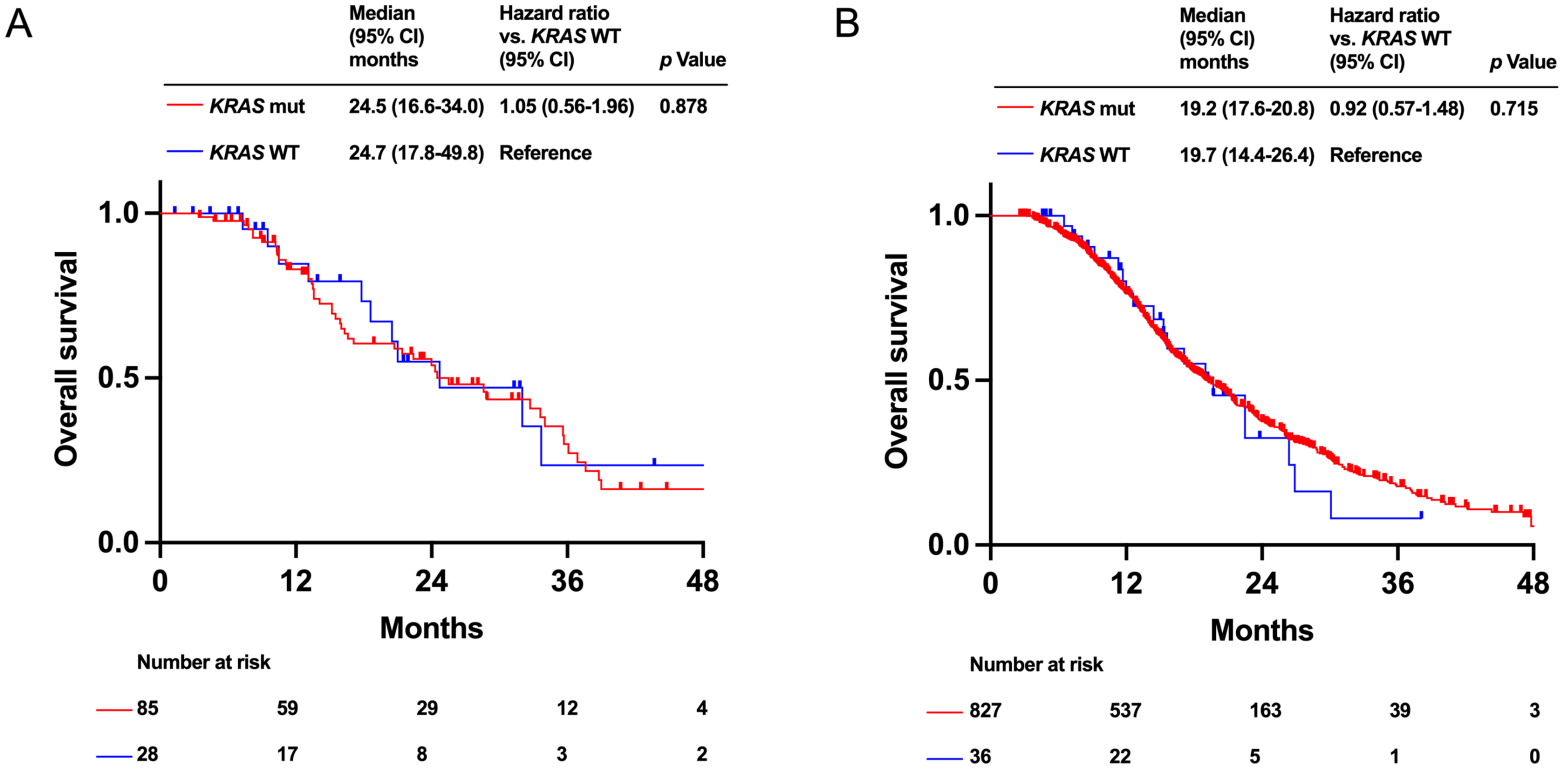
Kaplan–Meier analysis of overall survival (OS) by *KRAS* mutation status in PDAC patients with homologous recombination DNA repair (HRR) alteration group **(A)** and with HRR wild-type (WT) group **(B)**.

Regarding the presence or absence of *KRAS* mutations, OS was not significantly different between the HRR-altered (median OS: 24.5 months [95% CI: 16.6–34.0 months] vs. 24.7 months [95% CI: 17.8–49.8 months], *p* = 0.878) and HRR-WT groups (median OS: 19.2 months [95% CI: 17.6–20.8 months] vs. 19.7 months [95% CI: 14.4–26.4 months], *p* = 0.715) (Figures 5A,B).

**Figure 6:**
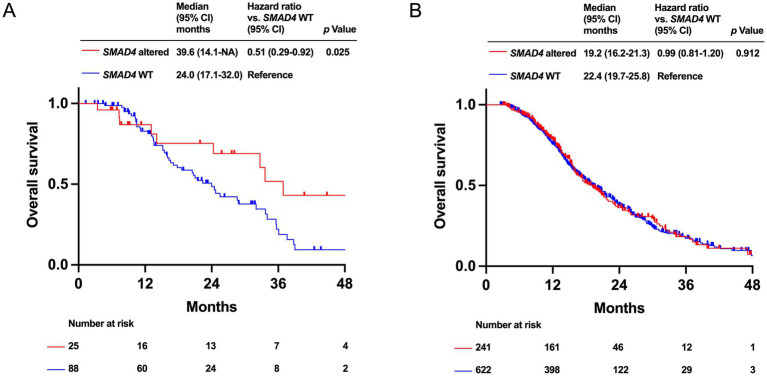
Kaplan–Meier analysis of overall survival (OS) by *SMAD4* alteration status in PDAC patients. Data for the homologous recombination DNA repair (HRR)-altered group **(A)** and the HRR-wild type (WT) group **(B)** are shown.

Patients with *SMAD4* alteration had a significantly better OS in the HRR-altered group (median OS: 36.4 months [95% CI: 14.1-NA months] vs. 24.0 months [95% CI: 17.1–32.0 months], *p* = 0.025). In the HRR-WT group, there is no significantly difference in OS between *SMAD4* alteration and *SMAD4* WT (median OS: 19.2 months [95% CI: 16.2–21.3 months] vs. 22.4 months [95% CI: 19.7–25.8 months], *p* = 0.912; Figures 6A,B).

Finally, we examined the prognostic factors associated with OS using univariate and multivariate analyses for the HRR-altered and HRR-WT groups, respectively (Supplementary Tables 3, 4). Therefore, these analyses were conducted including age, sex, and coexisting gene alterations. In HRR-altered group, *TP53* mutation was identified as a statistically significant independent predictor of OS (HR = 2.91; 95% CI 1.64–5.16, *p* < 0.001), while *SMAD4* alteration was associated with better OS (HR = 0.42; 95% CI 0.20–0.89, *p* = 0.023). In HRR WT group, *TP53* mutation (HR = 1.51; 95% CI 1.19–1.91, *p* < 0.001) and *CDKN2A* alteration (HR = 1.41; 95% CI 1.18–1.70, *p* < 0.001) were identified as a statistically significant independent predictor of OS.

## Discussion

The current study showed the prognostic significance of HRR status in a large Japanese PDAC cohort, showing that PDAC patients with HRR alteration had significantly longer TTF with the first-line FFX than PDAC patients with HRR-WT. In contrast, no survival benefit was observed with GA. Also, our study demonstrated the prognostic impact of coexisting *KRAS*, *TP53*, *CDKN2A*, and *SMAD4* alterations according to HRR status in PDAC.

Our results underscore previous findings of a correlation between HRR gene alterations and favorable prognosis, as well as the clinical benefit of platinum agents in PDAC (13, 17). Preclinical research has indicated that HRR gene-altered tumor cells have defective DNA repair, making them more vulnerable to platinum-induced DNA damage (24). The GENERATE trial demonstrated the superior survival benefit of GA over mFFX as a frontline treatment for locally advanced or metastatic PDAC patients in Japan (25). Therefore, GA is the preferred first-line treatment in Japan. The combination of irinotecan liposomes and fluoropyrimidine is preferred over mFFX following GA (26), limiting the subsequent use of mFFX and reduces platinum exposure throughout the clinical course. Although NGS is not currently recommended in the management guidelines for patients with PDAC (27), our findings highlight the importance of NGS in identifying patients with HRR-altered PDAC who are likely to benefit from platinum agents.

The current study demonstrated that *KRAS*, *TP53*, and *CDKN2A* mutations were significantly less prevalent in HRR-altered patients than in HRR-WT patients, consistent with previous findings (17, 18). The exact reasons for the differences in these gene mutations between HRR-altered and HRR-WT PDAC remain unclear. However, considering the carcinogenesis in *BRCA1/2*-mutated tumors (28), the development of HRR-altered PDAC may be different from the stepwise mutations of *KRAS*, *CDKN2A*, *TP53*, and *SMAD4* typically observed in general PDAC (29).

Although alterations in *TP53*, *CDKN2A*, *KRAS*, and *SMAD4* affect the prognosis of PDAC (3–6, 30), the influence of these gene alterations on the prognosis of HRR-altered PDAC has not been previously reported. To our knowledge, this study is the first to report on the prognostic impact of these gene alterations according to HRR alteration status in PDAC. The results demonstrated that *TP53* mutation was a poor prognostic factor, regardless of the HRR gene alteration status. Tumors harboring *TP53* mutations have higher genomic instability, resulting in the evasion of apoptosis, plasticity, and reduced lethality (31). Although *TP53* mutation is a well-known negative prognostic marker in PDAC (4, 6), our findings further confirm that is also associated with poor outcomes even in HRR-altered PDAC.

In the HRR-WT group, patients with *CDKN2A* alteration had significantly shorter OS than those with *CDKN2A* WT. In the HRR-altered group, patients with *CDKN2A* altered group had a trend toward worse prognosis than those with *CDKN2A* WT. These results were consistent with the previous studies (30, 32), while we indicated that *CDKN2A* alteration was a poor prognostic factor regardless of HRR alteration status in PDAC for the first time. The difference in prognosis between HRR-altered and WT group is probably due to the limited number of HRR-altered PDAC patients with *CDKN2A* alterations, despite our cohort being the largest in Japan, remains a challenge for this analysis. Understanding the status of *CDKN2A* alteration is important as *CDKN2A* loss frequently accompanies methylthioadenosine phosphorylase (*MTAP*) deletion (33) that is the target of AMG193, a protein arginine methyltransferase 5 (PRMT5) inhibitor (34).

The impact of *KRAS* mutation on survival outcomes has been inconsistent in previous studies (35, 36). However, our analysis found no significant differences in OS between patients with and without *KRAS* mutations in both the HRR-altered and WT groups. Although the prevalence of *KRAS* mutations was significantly lower in the HRR-altered group than in the HRR-WT group, it remains relatively high. As the development of pan-*KRAS* inhibitors (37), represents promising strategies to improve the prognosis of patients with PDAC, it is important to understand the presence and absence of *KRAS* mutations.

In the HRR-altered group, patients with *SMAD4* alteration had a significantly longer OS than those of *SMAD4* WT. Meanwhile, in the HRR-WT group, there was no significantly difference on survival between *SMAD4* altered and *SMAD4* WT groups. The impact of *SMAD4* alteration on survival outcomes has been obscure in previous study (5, 6). Our finding suggests a possibility that *SMAD4* alteration had a different prognostic impact according to HRR alteration status, while it is difficult to explain the reason at this point.

This study has some limitations. First, we could not differentiate between germline and somatic HRR gene alterations, and homologous recombination deficiency (HRD) status was not incorporated into the analysis as it was not reported by in the F1CDx testing. The HRD score, which serves as a genomic scar score and predicts treatment response to platinum agents in advanced PDAC, is generally higher in tumors with germline HRR alterations than in those with somatic alterations (38). Second, clinical data in the C-CAT database are limited with respect to both quantity and quality, with a notable number of missing entries. Thus, we were unable to perform propensity score matching to adjust for detailed patient characteristics when comparing the survival outcomes.

In conclusion, HRR gene alteration is a favorable prognostic factor and provides clinical benefit from platinum-based chemotherapy in PDAC. HRR-altered PDAC has a distinct molecular profile. Additionally, coexisting gene alterations affect the prognosis in both HRR-altered and HRR-WT PDAC patients. Collectively, these results emphasize the practical value of routine comprehensive genomic profiling to predict prognosis and guide treatment decisions in patients with PDAC.

## Data Availability

The data analyzed in this study is subject to the following licenses/restrictions: data are available upon reasonable request. Requests to access these datasets should be directed to Yusuke Kawanaka, 1954c0@med.kindai.ac.jp.
