# Supplementary material for: Prognostic impact of gene alterations via homologous recombination DNA repair gene alteration status in pancreatic ductal adenocarcinoma
Source: Front Med (Lausanne). 2025 Aug 25;12:1570731. doi: 10.3389/fmed.2025.1570731 (PMC12415010; doi:10.3389/fmed.2025.1570731)
Supplement: Supplementary file 1 [file Data_Sheet_1.PDF]

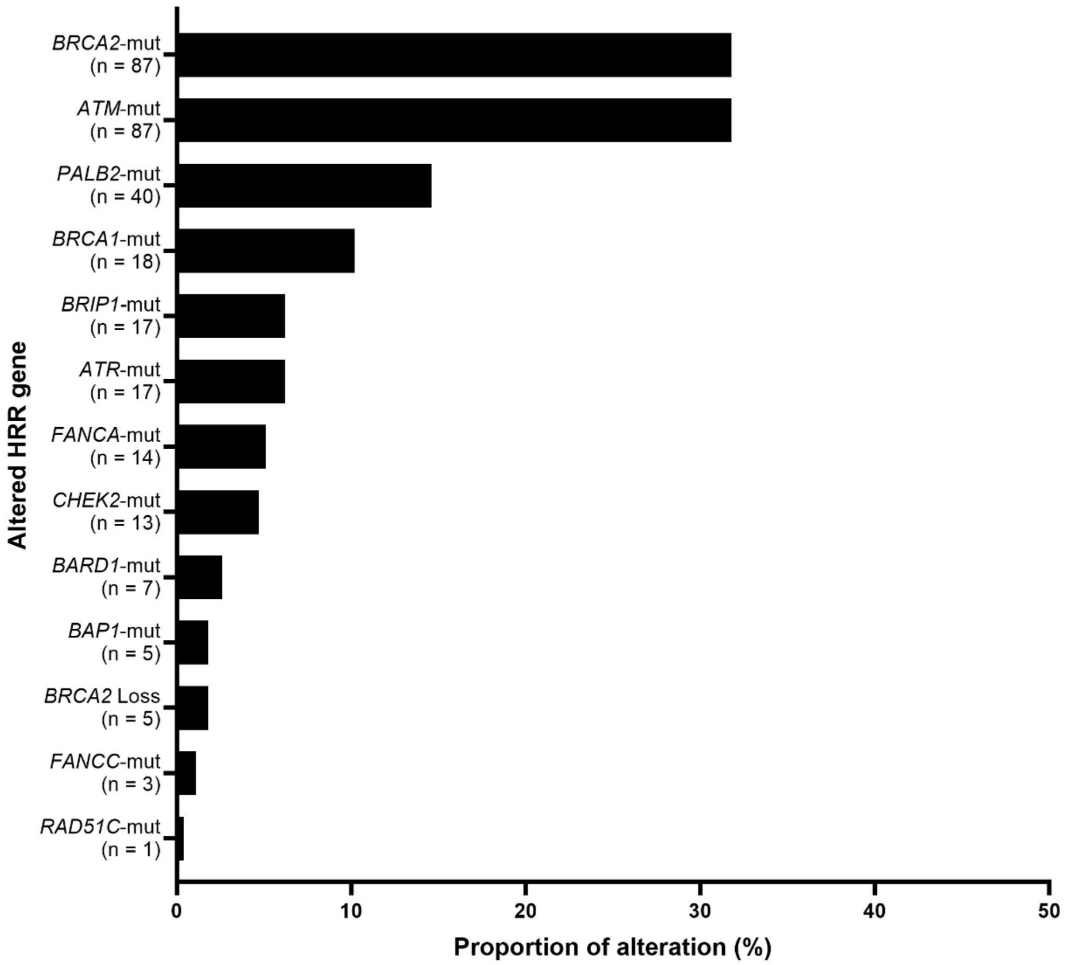

Supplementary Fig. 1

Frequency of each homologous recombination DNA repair (HRR) gene alteration in HRR-altered PDAC patients. There are 41 patients harboring two or more HRR gene alterations.

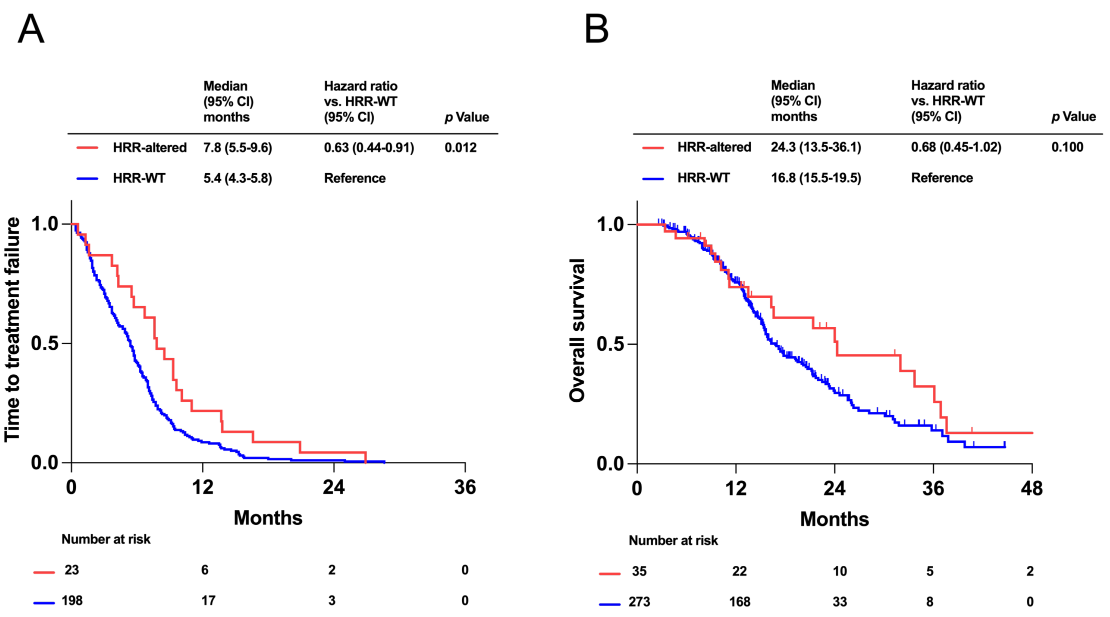

Supplementary Fig. 2A, B

Kaplan-Meier analysis of time to treatment failure (TTF) (A) and overall survival (OS) (B) based on homologous recombination DNA repair (HRR) gene alteration status in PDAC patients treated with folinic acid, fluorouracil, irinotecan hydrochloride, and oxaliplatin (FFX).

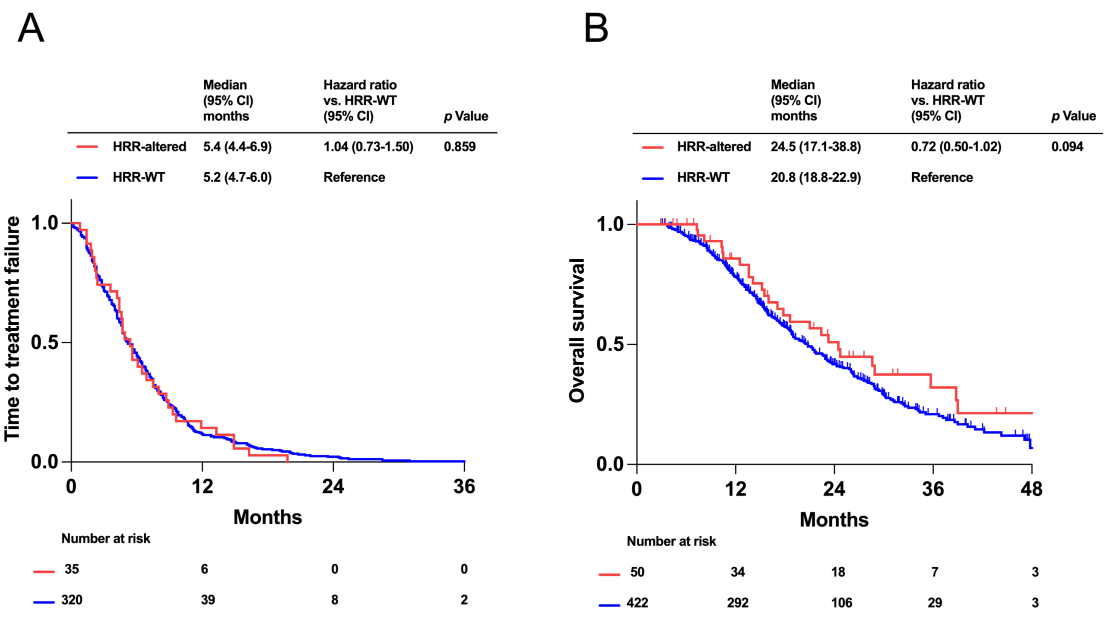

Supplementary Fig. 3A, B

Kaplan-Meier analysis of time to treatment failure (TTF) (A) and overall survival (OS) (B) based on homologous recombination DNA repair (HRR) gene alteration status in PDAC patients treated with gemcitabine and nab-paclitaxel (GA)

Supplementary Table 1. Patient characteristics by HRR gene alteration status in patients treated with palliative chemotherapy

Characteristic	HRR-altered group<br>n = 113	HRR-WT group<br>n = 863
Age at registration, years<br>Median (range)	65 (30-82)	66 (26-85)
Sex, n (%)		
Female	56 (49)	390 (45)
Male	57 (51)	473 (55)
Smoking, n (%)		
Yes	48 (42)	429 (50)
No	63 (56)	411 (48)
Unknown	2 (2)	23 (2)
Heavy alcohol consumption, n (%)		
Yes	11 (10)	106 (12)
No	96 (85)	674 (78)
Unknown	6 (5)	83 (10)
ECOG PS, n (%)		
0-1	111 (98)	832 (96)
≥ 2	1 (1)	20 (2)
Unknown	1 (1)	11 (1)
Metastatic sites, n (%)		
1	54 (48)	468 (54)
≥ 2	47 (42)	301 (35)
Unknown	12 (10)	94 (11)
Sampling methods, n (%)		
Surgery	27 (24)	212 (25)
Biopsy	85 (75)	630 (73)
Unknown	1 (1)	21 (2)

HRR: homologous recombination DNA repair; WT: wild type; ECOG PS: Eastern Cooperative Oncology Group performance status

Supplementary Table 2. Patient characteristics by HRR gene alteration status in patients treated with FFX or GA as first-line treatment

Characteristic	HRR-altered group n = 85	HRR-WT group n = 695
Age at registration, years Median (range)	65 (30-82)	66 (26-85)
Sex, n (%)		
Female	41 (48)	312 (45)
Male	44 (52)	383 (55)
Smoking, n (%)		
Yes	39 (46)	351 (51)
No	46 (54)	326 (47)
Unknown	0 (0)	18 (3)
Heavy alcohol consumption, n (%)		
Yes	10 (12)	86 (12)
No	71 (83)	553 (80)
Unknown	4 (5)	56 (8)
ECOG PS, n (%)		
0-1	84 (99)	677 (97)
≥ 2	0 (0)	12 (2)
Unknown	1 (1)	6 (1)
Metastatic sites, n (%)		
1	39 (46)	375 (54)
≥ 2	37 (44)	241 (35)
Unknown	9 (10)	79 (11)
Sampling methods, n (%)		
Surgery	19 (22)	156 (22)
Biopsy	66 (78)	534 (77)
Unknown	0 (0)	5 (1)
1st line chemotherapy regimen, n (%)		
FFX	35 (41)	273 (39)
GA	50 (59)	422 (61)
2nd line chemotherapy, n (%)		

FFX	14 (16)	84 (11)
GA	16 (19)	136 (20)
Other	24 (28)	241 (35)
Unknown	31 (36)	234 (34)

HRR: homologous recombination DNA repair; WT: wild type; ECOG PS: Eastern Cooperative Oncology Group performance status; FFX: folinic acid, fluorouracil, irinotecan hydrochloride, and oxaliplatin; GA: gemcitabine and nab-paclitaxel

Supplementary Table 3. Univariate and multivariate analyses for OS in HRR-altered patients.

	Univariate Analysis			Multivariate Analysis		
	HR	95% CI	p Value	HR	95% CI	p Value
Age (>70 vs. ≦70, y.o.)	1.07	0.60-1.91	0.816	1.02	0.54-1.91	0.962
Sex (Male vs. Female)	0.82	0.48-1.39	0.461	0.70	0.41-1.20	0.204
TP53 (mutation vs. wild type)	2.57	1.51-4.37	< 0.001	2.91	1.64-5.16	< 0.001
CDKN2A (alteration vs. wild type)	1.35	0.81-2.25	0.095	1.71	0.99-2.99	0.130
KRAS (mutation vs. wild type)	1.05	0.56-1.96	0.878	0.95	0.48-1.90	0.891
SMAD4 (alteration vs. wild type)	0.51	0.29-0.92	0.025	0.42	0.20-0.89	0.023

OS: overall survival; HRR: homologous recombination DNA repair

Supplementary Table 4. Univariate and multivariate analyses for OS in HRR-wild type patients.

	Univariate Analysis			Multivariate Analysis		
	HR	95% CI	p Value	HR	95% CI	p Value
Age (>70 vs. ≦70, y.o.)	0.97	0.81-1.17	0.483	0.95	0.79-1.14	0.582
Sex (Male vs. Female)	1.04	0.87-1.24	0.646	1.01	0.85-1.21	0.852
TP53 (mutation vs. wild type)	1.54	1.26-1.89	< 0.001	1.51	1.19-1.91	< 0.001
CDKN2A (alteration vs. wild type)	1.27	1.06-1.53	< 0.001	1.41	1.18-1.70	< 0.001
KRAS (mutation vs. wild type)	0.92	0.57-1.48	0.715	0.83	0.52-1.32	0.431
SMAD4 (alteration vs. wild type)	0.99	0.81-1.20	0.912	0.95	0.78-1.16	0.626

OS: overall survival; HRR: homologous recombination DNA repair
